# Influence of Prolonged Dental Bleaching on the Adhesive Bond Strength to Enamel Surfaces

**DOI:** 10.1155/2020/2609359

**Published:** 2020-05-14

**Authors:** Juliana C. P. Baia, Roberta P. Oliveira, Mara E. S. Ribeiro, Rafael R. Lima, Sandro C. Loretto, Mário H. Silva e Souza Junior

**Affiliations:** ^1^Department of Restorative Dentistry, UFPA-Federal University of Pará, Belém 66075-110, Brazil; ^2^Laboratory of Functional and Structural Biology, UFPA-Federal University of Pará, Belém 66075-110, Brazil

## Abstract

The objective of this in vitro study was to assess the influence of prolonged bleaching pre- and postrestoration on the bond strength (microshear) to enamel using 4% hydrogen peroxide (PH4). In the postrestorative bleached specimens evaluation, the composite cylinders were assembled after bleaching, while in the prebleached specimens, the cylinders were assembled before. Therefore, in the postbleached specimens, 60 bovine teeth were randomly assigned as follows: G1: control; G2: 14 days bleaching before bond strength (BS) testing; G3: 21 days; and G4: 28 days. In prebleached specimens, 180 bovine teeth were randomly assigned as follows: G1: control; G5: 14 days bleaching, storage in artificial saliva (AS) for 24 h before BS testing; G6: 14 days beaching, AS storage for 7 days before BS testing; G7: 21 days bleaching, AS storage for 24  h before BS testing; G8: 21 days bleaching, AS storage for 7 days before BS testing; G9: 28 days bleaching, AS storage for 24 hours before BS testing; and G10 : 28 days bleaching, AS storage for 7 days before BS testing. The results were submitted to ANOVA one-way (postrestoration bleaching) and two-way (prerestoration bleaching) and Tukey's post hoc test (*p* ≤ 0.05). In the postrestoration bleaching, no statistical difference between times was found. However, when bleached groups were compared to the control (G1), an expressive difference was detected (*p* ≤ 0.0001). For prerestoration bleaching, all experimental groups were statistically different from G1 (*p* ≤ 0.05), except G6 (*p* ≥ 0.01), and for G5 and G6, statistical differences were found (*p* ≤ 0.01). There were no significant differences between G7 and G8 and between G9 and G10, regardless of the AS storage times (*p* ≥ 0.05). It was concluded that prolonged bleaching with PH4 decreased adhesion resistance regardless of the moment of the bleaching (post- and prerestoration bleaching).

## 1. Introduction

Dental bleaching treatments provide fast and expressive results without wearing dental structure away. These facts, associated with the desire for a brighter and more attractive smile, made this treatment quite popular, increasing its demand in the last decades. [1, 2] When hydrogen peroxide dental bleaching gels are activated, they release oxidizing agents, which can penetrate the enamel and dentin ultrastructures, producing a chemical reaction. The hydrogen peroxide, under alkaline conditions, undergoes ionic dissociation, giving rise to hydroxyl anions (OH-), a highly unstable free radical [3]. The double bonds involving carbon atoms and the chemical elements nitrogen and oxygen (present in the pigments of the dental structure) are very susceptible to electron donators and represent the main target of the action of the peroxide. The double bonds are present in the organic molecules and are responsible for the color darkening. Once the double bonds are broken, the free radicals change the molecule's absorption energy, thus the light-matter interaction, bleaching the tooth [4].

Since many patients have composite restorations and need dental bleaching therapies, it is important to consider that bleaching may affect the physical and chemical properties of restorative materials, including roughness and hardness, generate cracks and marginal degradation, release metallic ions, and finally, decrease bond strength to dental structures [5–8]. The surrounding enamel may also be affected during bleaching, compromising the integrity of the interface adhesion [9, 10]. Conversely, when a patient needs restoration after bleaching, concerns arise on the effects of peroxides inside the dental substrates. Depending on the concentration of the bleaching agent, the bond strength to enamel decreases by approximately 60% when compared to unbleached teeth. This reduction seems to be related to trapped residual oxygen, which interferes with the infiltration of the bonding monomers, the subsequent reduction in length and number of resin tags, and the inhibition of polymerization reaction. Therefore, a delay of one to three weeks is recommended before the adhesive restoration procedures take place [11–14].

The literature has shown that the addition of fluorides and calcium to bleaching agents reduces enamel susceptibility to erosion and caries [15–17], preserves its mechanical strength, and reduces the mineral loss during the treatment without influencing the effectiveness of the dental bleaching process [9, 18]. Searching for more expressive results, some patients extend the use of dental bleaching, especially in at-home treatment. This prolonged use may overcome the so-called “saturation point.” This is the point at which the dental bleaching gels no longer act only in the chromogens, but they will negatively affect the mineral composition, resulting in chemical and mechanical property alterations of enamel [19, 20]. Thus, the aim of this study was to verify the influence of prolonged dental bleaching protocols in the bond strength to dental enamel, in two different moments: post- and prerestoration bleaching using 4% hydrogen peroxide with calcium.

## 2. Materials and Methods

This study was approved by the local Animal Research Ethics Committee (CEUA/4776201016). A total of 240 sound bovine incisor teeth were used. The inclusion criteria were as follows: the teeth should be erupted in the oral cavity, present no crack or fracture, and have completed root formation.

The teeth were disinfected in a 0.1% thymol solution for one week. After that, they were washed in running tap water to remove any remaining blood or tissues. Next, all teeth were analyzed with a stereoscopic microscope (40x) to detect cracks or fractures. The selected teeth were then stored in distilled water under refrigeration (4°C) (ISO TS 11405 : 2003).

The teeth were sectioned at the cementum-enamel junction (CEJ), using a double-sided diamond disc (KG Sorensen, Cotia, São Paulo, SP, Brazil). The root portions were discarded. The dental crown was embedded with self-cured acrylic resin (Auto Clear, DentBras, Pirassununga, SP, Brazil) into polyvinylchloride rings (20 mm in diameter and 13 mm high). The enamel surfaces were positioned 1 mm above the resin surface to avoid an acrylic smear layer on the testing substrate. Therefore, after 24 h, the enamel surfaces were ground wet using silicon carbide discs (#180, 400, and 600) in a horizontal polisher (Aropol VV-PUD, Arotec, Cotia, SP, Brazil). Next, they were ultrasonically washed (TD30 Plus; Bio-Art, SP, Brazil) in distilled water for 20 minutes and analyzed with a stereomicroscope (40x) to ensure the absence of dentin exposure and cracks. Finally, the embedded enamel blocks were randomly assigned (Bioestat Software 5.0®) in two bleaching moments: postrestoration (*n* = 60) and prerestoration (*n* = 180) (Table 1).

It was necessary for this study to set reliable indicators to obtain based and reproducible outcomes. Thus, *n* = 10 was determined for sample size calculation, where the minimal desired difference between the control group (14 days bleached with PH4) and the experimental extended bleaching groups was 20%. The statistic power was 80% (*α* = 5%), which was determined by the bilateral test. A sample size of *n* = 30 was used for each experimental group.

### 2.1. Postrestoration Bleaching

**Figure 1:**
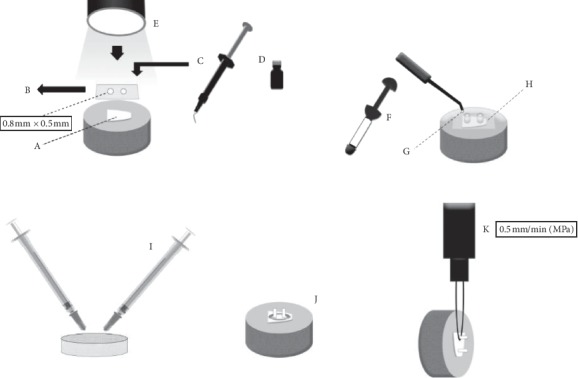
A and B: acid-resistant double-sided tape perforated by rubber sheet punch (0.8  mm diameter), delimiting the adhesive bond area to the enamel. C: conditioning with 35% phosphoric acid (Adper Scotchbond 3M ESPE, Sumaré, SP, Brazil) for 30 seconds; gel removal with an air–water spray for 30 seconds; drying with air jets. D: application of the adhesive system according to the manufacturer (Adper Single Band 2, 3M ESPE, Sumaré, SP, Brazil). E: photoactivation for 20 seconds with an LED-type photoactivation device (Ultrablue D-2000. DMC, São Carlos, SP, Brazil), (900  mW/cm^2^). F, G, and H: acid-resistant tape first layer removal; Tygon® pipes placement, 0.8  mm internal diameter and 0.5  mm high, coinciding with tape demarcations. Pipes filled with Filtek Z350 XT composite resin (3M ESPE, Sumaré, SP, Brazil) and photoactivation for 40 seconds. Two cylinders of composite resin were made in each tooth. After 24 h of storage in distilled water (37°C), removal of Tygon® pipes and the second layer of tape with the aid of scalpel blade n 12. I and J: 4% hydrogen peroxide with calcium (White Class with 4% calcium; FGM, Santa Catarina (SC), Brazil), using a 0.1 mL proportion of bleaching gel for 0.05 mL of artificial saliva, and conditioned during the bleaching process (2 (h), in a biological stove (37°C). Application delimitation of bleaching agent in 2 mm distance around the adhesive interfaces using a gingival barrier (TOP DAM, FGM, SC, Brazil). After bleaching gel was applied, specimens were washed using an air–water spray at approximately 5 cm from enamel surface, for 1 minute, and the gingival barrier was removed. Between sessions, specimens were stored in AS and conditioned in a biological stove (37°C). The gel was applied daily, according to the described duration for each group (Table 1). K: at the end of the dental bleaching systems for each group, samples were attached to a universal testing machine for the microshear test (Kratos Equipment's LTDA, Cotia, SP, Brazil).

The enamel surfaces were polished, the adhesive area was delimited, and 02 composite resin cylinders were built up on each enamel block, according to the schematic shown in Figure 1. Next, the samples were divided into 04 groups, according to the number of bleaching procedures (Table 1), and the microshear bond strength tests were performed (Figure 1). The microshear bond strength results presented normal distribution (Shapiro–Wilk test) and were submitted to one-way ANOVA and Tukey's post hoc tests (*p* ≤ 0.05).

### 2.2. Prerestoration Bleaching

**Figure 2:**
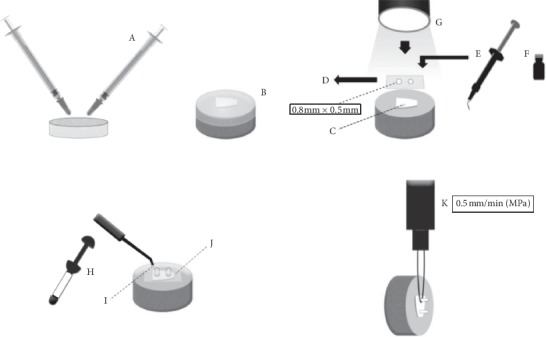
A: 4% hydrogen peroxide with calcium (White Class with 4% calcium; FGM, SC, Brazil) (PH4), using a 0.1 mL proportion of bleaching gel for 0.05  mL of AS, and sample packaging, during the bleaching process 2 h in a biological stove (37°C). B: placement of the customized acetate trays proportioned with PH4 and AS. C and D : acid-resistant double-sided tape perforated with rubber sheet punch (0.8 mm diameter), delimiting the enamel adhesive bond area. E: conditioning with 35% phosphoric acid (Adper Scotchbond 3M ESPE, Sumaré, SP, Brazil) for 30 seconds; gel removal with an air–water spray for 30 seconds, drying with air jets. F: application of adhesive system according to manufacturer's recommendations (Adper Single Bond 2, 3M ESPE, Sumaré, SP, Brazil). G: photoactivation using an LED-type photoactivation device with 900 mW/cm^2^ (Ultra Blue D-2000. DMC, SP, Brazil). H, I, and J: acid-resistant tape first layer removal; Tygon® pipes placement, 0.8 mm diameter and 0.5 mm high, coinciding to tape demarcations. Filling of the pipes with Filtek Z350 XT composite resin (3M ESPE, Sumaré, SP, Brazil) and photoactivation for 40 seconds. Two cylinders of composite resin were made in each tooth. After 24 h of storage in distilled water (37°C), removal of Tygon® pipes, and the second layer of tape with the aid of scalpel blade n°12. The resin cylinders were examined with a stereoscopic microscope (40x). K: at the end of the dental bleaching systems in each group, samples were attached to a universal testing machine for the micro-shear test (Kratos Equipment's LTDA, Cotia, SP, Brazil).

The enamel blocks were divided into 07 groups according to the number of bleaching procedures (Table 1). After all the bleaching procedures, 02 composite resin cylinders were built up on each bleached enamel surface (Figure 2). The results were submitted to two-way ANOVA test and Tukey's post hoc test (Bioestat Software 5.0®) (*p* ≤ 0.05).

After bond strength tests, the fractured specimens (post- and prebleached) were analyzed with a stereoscopic microscope (40x) (SZ2-ILST, Olympus SZ61, Tokyo, Japan) to determine the fracture patterns as follows: adhesive, cohesive (in enamel or composite resin), or mixed. After the fracture pattern evaluation, three specimens of each group were randomly selected for a SEM evaluation at 50x magnification (LEO-1430; Carl Zeiss, Oberkochen).

## 3. Results

### 3.1. Postrestoration Bleaching

The highest mean was observed in G1 (20.87  MPa) and the lowest in G2 (13.59  MPa). No statistical difference was found among any bleached groups, except when compared to G1 (negative control/no dental bleaching) (*p* ≤ 0.0001). Thus, the dental bleaching decreased enamel adhesive bond strength, regardless of the number of bleaching gel applications (14, 21, or 28 days) (Table 2).

**Figure 3:**
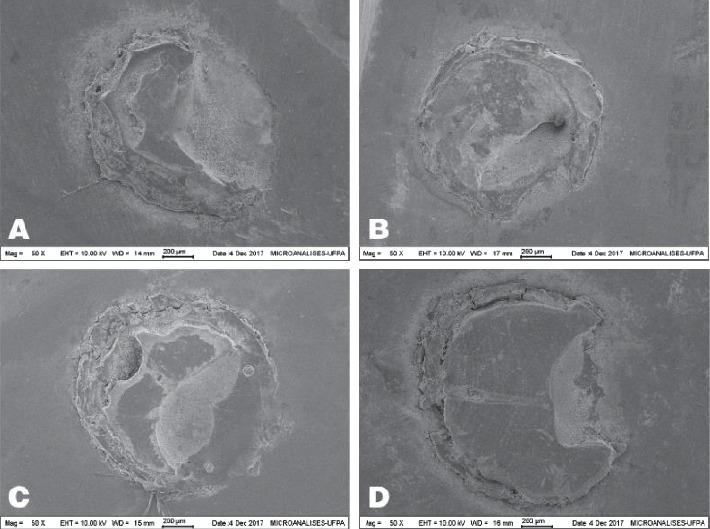
(a) Mixed fracture pattern in G1; (b) mixed fracture pattern in G2; (c) mixed fracture pattern in G3; (d) mixed fracture pattern in G4.

The most prevalent fracture pattern for all experimental groups was the mixed type (76%), followed by the adhesive type (24%) (Figure 3).

### 3.2. Prerestoration Bleaching

The highest mean was observed in G1 (20.87 MPa) and the lowest in G5 (13.64 MPa). All bleached groups were statistically different from the negative control group (G1) (*p* ≤ 0.05), except G6, which received bleaching gel application as recommended by the manufacturer (14 days) and stored in AS for 7 days (*p* > 0.05). Statistical differences were found (*p* ≤ 0.01) when G5 (14 days/24 h in AS) and G6 (14 days/07 days in AS) were compared. For all groups, the specimens were stored for 24 h in AS (G5, G7, and G9), and there was no statistical difference in bond strength values (*p* ≥ 0.05) (Table 3).

**Figure 4:**
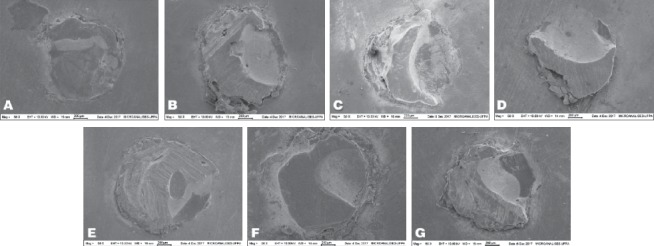
Mixed fracture pattern in (a) G1; (b) G2; (c) G3; (d) G4; (e) G5; (f) G6; (g) G7.

When dental bleaching was performed for 21 days (G7 and G8) and 28 days (G9 and G10), no statistical difference was found; therefore, the AS storage time had no influence on BS (*p* ≥ 0.05) (Table 3). The most prevalent fracture pattern for all experimental groups was the mixed type (80%), followed by the adhesive one (20%) (Figure 4).

## 4. Discussion

### 4.1. Postrestoration Bleaching

In this part of the study, only the bleaching gel White Class (4% HP + calcium) was applied on specimens (composite cylinder bonded to enamel surface). The bleaching gel was applied for 2 hours each day for 14 days (manufacturer's instructions), 21 days, and 28 days. Prolonging the bleaching gel application beyond the manufacturer's recommendation did not cause any significant differences in the bond strengths of all the bleached groups. There was a significant difference, however, when the bleached groups were compared to the control, nonbleached group. It has been proved that bleaching decreases bond strength when applied to adhesive interfaces. A previous study Barcellos et al. (2010) [17] applied different concentrations of carbamide peroxide gel (10%, 15%, and 20%), following the manufacturer's instructions, on enamel and dentin adhesive interfaces. On enamel, concentrations of 15% and 20% reduced the bond strength [17]. It was speculated that the weakening mechanism of these interfaces when bleaching gels are used is due to the attack on the surrounding enamel, changing its ultramorphological structures [21], which may contribute to the degradation process.

Enamel mineral loss due to a significant decrease in calcium and phosphate content may occur after bleaching, which may even increase the susceptibility of enamel to demineralization. In that case, porosity in the enamel created by the bleaching agent may have acted as a stress raiser, resulting in premature failures. Moreover, during bleaching, hydrogen peroxide breaks down into free radicals, which may induce oxidative cleavage of polymer chains and then lead to chemical softening of the dental material. Consequently, the free radicals may affect the resin-filler interface and cause filler-matrix debonding, leading to the formation of microscopic cracks and increasing surface roughness [9, 22].

In the formation of the acquired film, the natural saliva acts to create a protective barrier for the teeth, and it limits their contact with acid substances from the diet. This prevents the reduction of dental hardness and regulation of calcium (Ca), phosphate, and salivary proteins [23, 24]. So, the saliva's ability to reinforce and replace the lost minerals on bleached enamel demonstrates that the loss of chemical components after the dental bleaching is naturally controlled by saliva components and by fluorinated solutions [24].

The Ca present in the bleaching gel aims to minimize the mineral losses caused by the hydrogen peroxide action, preventing structural losses, higher microhardness, and less superficial roughness to enamel after the dental bleaching and submission to acidic challenges, when compared to the gel without the addition of Ca in its composition [25, 26].

Considering the abovementioned statements, it was expected that saliva and mineral content in the bleaching gels could reverse the eventual damage caused during bleaching treatments. However, these expected events were not verified in the present study. In all the groups with bleached specimens, regardless of the number of treatment applications and AS storage regimes, there was a drop in bond strength, when compared to the control (nonbleached) group.

In the same line of study, that is, to assess the influence of enamel mineral replacement, Cavalli et al. (2012) evaluated the influence of fluoride containing carbamide peroxide and fluoride containing adhesive associated with pH cycling [9]. The aim was to evaluate whether the remineralizing agents could protect these interfaces from degradation. That study also detected a reduction in bond strength in the bleached experimental groups when compared to the nonbleached groups (control), regardless of the presence of fluoride. In the gel composition used in the present study, sodium fluoride and calcium were included as remineralizing agents. The results were similar to [9], that is, these components could not prevent the reduction of bond strength of bleached interfaces, as well as the storage in AS.

Some studies [9, 18] have demonstrated that the type of adhesive system used during the restoration process may influence the bonding deterioration process, so from this point of view, three-step adhesive systems seem to be advantageous. This is likely due to the presence of a less hydrophilic layer (bonding agent), which might act as a protective shield against the infiltration of the bleaching gel into the adhesive interface. In Cavalli et al.'s study [9], the interfaces obtained with the three-step adhesive system presented less reduction of bond strength, compared to two-step adhesives. In the present study, the reduction in the bond strength, regardless of the length of gel exposure (14, 21, or 28 days), may be explained in part by the adhesive strategy employed during the assembly on the composite cylinders. The two-step adhesive system (Adper Single Bond 2) presents high hydrophilicity and may have been one of the reasons for the infiltration of the gel components into the adhesive interface. Indeed, in the study of Dudek (2013) [18], besides a two-step adhesive system, mild and strong self-conditioning systems were applied. The results showed that when a strong (more acidic) self-etching adhesive system was used, there was a negative influence on the bond strength of the adhesive interfaces. Therefore, it is possible to consider that the higher the hydrophilicity, the less protected is the interface to the moisture environment.

Therefore, considering that bleaching procedures may compromise the bonded interfaces of existing restorations, and it would be prudent to advise that, after bleaching procedures when composite restorations are present, a surface repair, for resealing purposes, should be done.

### 4.2. Prerestoration Bleaching

Some studies [14, 27, 28] have proved that waiting between 7 and 21 days is necessary to neutralize the oxidizing effects of bleaching gels on tooth structures. Therefore, in this part of the study, the influences of different times of gel exposure and AS storage prior to the bonding procedures were evaluated. The results demonstrated a reduction in bond strength when storage time was 24 h, regardless of the bleaching protocol (14, 21, or 28 days). On the other hand, a 7-day AS storage time was able to recover the bond strength only when the 14-day bleaching protocol was used, which is the manufacturer's instruction. Instead, the recovery of bond strength values was not observed for the prolonged bleaching groups (21 or 28 days).

Considering the abovementioned information, some concerns arise about bleaching procedures, particularly when adhesive techniques must be carried out. The presence of residual oxygen in the dental structure inhibits resin polymerization. Therefore, the quality of the hybrid layer may be affected. It has been shown, however, that this phenomenon is time-dependent (transient) [6, 17, 29]. Other types of enamel damage were observed, such as erosion, decreased hardness, and chemical and morphological alterations [30–32]. Therefore, when the bleaching time is extended, these changes may be exacerbated. In SEM observations, it is possible to detect an increase in the exposure of enamel rods and the dissociation of some chemical elements, such as calcium and phosphorous. These changes are directly related to the time that the substrate is exposed to the bleaching gels [14, 22, 33, 34]. Moreover, the morphological aspect of the enamel bleached for longer periods was comparable to Silverstone's Type III etching pattern. This pattern is characterized by larger areas and paucity of exposed interprismatic enamel, which is unfavorable to the adhesive bonding mechanism [22, 35].

When bleaching procedures were carried out according to the manufacturer's instructions (14 days) followed by AS storage for 24 h (G5), a bond strength of 13.64 MPa was achieved. This result, when compared to that observed for the unbleached group (20.87 MPa), confirmed that the hydrogen peroxide treatment reduced the adhesion to enamel by approximately 35%. This finding is consistent with other studies [26, 36, 37] which have demonstrated that 24 hours is not enough to complete oxygen elimination. The reduction in bond strength might lead to adhesion failure, and under a clinical standpoint, it is crucial, since a restoration after bleaching procedures is often necessary.

When 14 days bleaching was carried out followed by a 7-day AS storage, the enamel bond strength was recovered to the results observed for the unbleached group (G1). On the other hand, when bleaching time was extended to 21 (G7 and G8) or 28 days (G9 and G10), the bond strength did not return to the level of the control group, regardless of the AS storage time. Thus, one can speculate that under extended bleaching protocols, the 7-day AS storage time was not enough to eliminate the greater amount of residual oxygen trapped in the enamel structure [7, 28, 36, 38]. In a recent study by our group, a postbleaching waiting time longer than 7 days was suggested when enamel was exposed to a prolonged bleaching procedure [14].

It has often been observed clinically that the desired color was not achieved after 14 days of bleaching, so the at-home bleaching was extended. Indeed, at times it is performed in association with in-office bleaching. According to the results of the present study, a 7-day AS storage was not enough to recover the initial bond strength when the bleaching time was extended. In the literature, waiting times up to 28 days are mentioned as necessary before restoration. Nevertheless, waiting 21 days to restore bleached teeth is not always feasible for different reasons [14, 22]. Beyond that, there are no specific rules for waiting times associated with these different protocols (at home, in office, or a combination of the two). Other studies have mentioned the possibility of using antioxidizing agents, such as sodium ascorbate, to reduce the waiting time before restoration [27, 28]. Considering the great variety of methods in this field of investigation, it is difficult to establish specific waiting times for different bleaching protocols. Therefore, according to the results of the present study, when the time recommended by the manufacturer is exceeded, an increase in the waiting time before restoration should be considered [17, 32].

There is a controversy about the effect of delaying the restoration process on the bond strength of bleached teeth. Some have reported that 24 h would be enough to achieve adequate adhesive value after dental bleaching with 10% carbamide peroxide, while others have suggested that a longer exposure time (between 7 and 14 days) is necessary before performing the adhesive protocol [7, 10, 28]. In a systematic review, it has been shown that delaying any restoration process for one week is as effective as using antioxidizing agents [28].

Simulating some dental bleaching protocols for a prolonged time, Souza et al. [39] verified several ultramorphological and chemical alterations on enamel. These included the exposure of several enamel prisms and a considerable decrease in calcium and phosphorus. Furthermore, by simulating the prolonged dental bleaching effect on the microhardness and superficial roughness using both professional and homemade bleaching agents freely available to the patients, there was a gradual increase in dental roughness as the treatment progressed, until it ended after eight weeks, while there were no changes in the enamel's physical characteristics, and natural saliva could not reduce the microhardness [34].

In 2019, Vilhena et al. [21] showed that the abusive use of bleaching gel for prolonged periods (21 and 28 days) led to enamel organic matrix degradation after 21 days. At that time, variations in Ca and P values were observed through EDS. Microhardness and roughness properties were also altered. Conversely, through X-ray diffraction, the crystalline structure of hydroxyapatite was not modified, even after the treatment time suggested by the manufacturer. It is important to remark, however, that other sources of stress may be present during bleaching procedures, such as brushing and diet. Still, in that mentioned study, the storage in AS between experimental trials could not revert the changes. Another study [14] could not demonstrate that the presence of Ca in the bleaching gel compositions could not minimize the mechanical damage on the enamel surfaces, previously mentioned.

Therefore, the prolonged and continuous contact of bleaching gels on dental structures during clinical treatments associated with routine human practices may cause irreversible damage, even when low peroxide concentrations are used. Therefore, dental professionals must emphasize to their patients the need to obey the instructions of the manufacturers and attend the supervising appointments to reach an esthetic result without damaging the dental structures [33, 40].

## 5. Conclusions

When the adhesive interface is established prior to dental bleaching, regardless of the gel exposure times, the bond strength to enamel decreased. When dental bleaching occurs before the adhesive procedures, prolonged bleaching times (21 and 28 days) led to a significant drop in the strength of the enamel bond regardless of the storage period (24 h or 7 days).

## Figures and Tables

**Table 1:** Description of experimental groups.

Postrestoration bleaching		Prerestorative bleaching		
Groups	Bleaching days	Groups	Bleaching days	Storage time
G1	—	G1	—	—
G2	14	G5	14	24 h
G3	21	G6	14	7 days
G4	28	G7	21	24 h
		G8	21	7 days
		G9	28	24 h
		G10	28	7 days

^*∗*^Sample size calculated after the pilot test.

**Table 2:** Postrestoration bleaching results of the adhesive bond strength (MPa) to enamel surfaces.

	Experimental groups			
	G1	G2	G3	G4
Mean	20.876^A^	13.599^B^	14.029^B^	16.549^B^
(Standard deviation)	(±5.14)	(±3.91)	(±5.36)	(±5.88)

**Table 3:** Prerestoration bleaching results of the adhesive bond strength (MPa) to enamel surfaces.

	Experimental groups						
	G1	G5	G6	G7	G8	G9	G10
Mean (Standard deviation)	20.876	13.648^A,a^	8.564^B,b^	14.816^C,a^^*∗*^	16.445^C;b,c^^*∗*^	13.850^D,a^^*∗*^	13.932^D,c^^*∗*^
	(±5.14)	(±4.13)	(±6.87)	(±4.54)	(±5.76)	(±5.22)	(±5.66)

Distinct capital letters indicate statistical difference between the same period of dental bleaching; distinct lowercase letters indicate statistical difference between the same period of storage in AS. ^*∗*^Statistical difference compared with the unbleached group (G1).

## Data Availability

The data obtained and used to support the conclusions of this study are included in the article.
